# Multiscale Contextual Poverty in the Netherlands: Within and Between-Municipality Inequality

**DOI:** 10.1007/s12061-021-09394-3

**Published:** 2021-06-01

**Authors:** Ana Petrović, David Manley, Maarten van Ham

**Affiliations:** 1 Delft University of Technology, Delft, The Netherlands; 2 University of Bristol, Bristol, UK; 3 University of St Andrews, St Andrews, UK

**Keywords:** Contextual poverty, Spatial scale, Spatial inequality, Distance profile, Exposure, Theil index

## Abstract

Contextual poverty refers to high proportions of people with a low income in a certain (residential) space, and it can affect individual socioeconomic outcomes as well as decisions to move into or out of the neighbourhood. Contextual poverty is a multiscale phenomenon: Poverty levels at the regional scale reflect regional economic development, while meso-scale concentrations of poverty within cities are related to city-specific social, economic and housing characteristics. Within cities, poverty can also concentrate at micro spatial scales, which are often neglected, largely due to a lack of data. Exposure to poverty at lower spatial scales, such as housing blocks and streets, is important because it can influence individuals through social mechanisms such as role models or social networks. This paper is based on the premise that sociospatial context is necessarily multiscalar, and therefore contextual poverty is a multiscale problem which can be better understood through the inequality within and between places at different spatial scales. The question is how to compare different spatial contexts if we know that they include various spatial scales. Our measure of contextual poverty embraces 101 spatial scales and compares different locations within and between municipalities in the Netherlands. We found that the national inequality primarily came from the concentrations of poverty in areas of a few kilometres, located in cities, which have different spatial patterns of contextual poverty, such as multicentre, core-periphery and east–west. In addition to the inequality between municipalities, there are considerable within-municipality inequalities, particularly among micro-areas of a few hundred metres.

## Introduction

Over the last three decades, socioeconomic inequalities in European cities have been growing, and this has led to increasing spatial concentrations of people with low income in certain (residential) areas (Tammaru et al., 2016). Living in neighbourhoods with concentrated poverty can affect the socioeconomic outcomes of people, such as their education and labour market performances (Van Ham et al., 2012); moreover, it can influence individual decisions to move into or out of the neighbourhood (Bolt & Van Kempen, 2003; Sampson et al., 2002; Van Ham & Clark, 2009). Contextual poverty can emerge at the scale of streets, or housing blocks, collections of streets in the inner-city neighbourhoods or suburbs, often known as ‘neighbourhoods’, or even across regions. This makes contextual poverty a *multiscale* problem which is related to both the causes and consequences of poverty. Multiple factors, such as economic and housing structures, can lead to concentrations of poverty at different spatial scales from region to neighbourhood. In turn, poverty at various spatial scales can affect individual outcomes of people through a variety of contextual effects mechanisms (see Sampson et al., 2002; Galster, 2012), including social mechanisms at a smaller scales and stigmatisation at larger ones (Petrović et al., 2019). So, with increasing scale, there are new contexts introduced at which poverty expresses itself spatially, and at which individual outcomes are affected.

Even within countries which have relatively low levels of poverty, there are regional differences, where some parts of the country are poor compared with other parts and regional inequality is generally multiscalar (Wei, 2015). Below the regional scale, some neighbourhoods in cities or towns are poorer than others. In fact, there are inequalities at different levels of the urban system, both between and within cities, and at different spatial scales. Inequalities at different spatial scales contribute to national level inequalities, as well as to people’s individual exposure to their spatial context. Surprisingly, the literatures on global inequality, segregation and neighbourhood effects, which are all concerned with contextual poverty, rarely start from the premise that sociospatial context is necessarily multiscalar. Instead, analyses are often carried out using a single scale, often drawing on readily available administrative spatial units. Using a single scale can neglect important spatial context effects at other scales, and we argue that spatial inequality and its effects cannot be fully understood by simply taking one arbitrary scale available in the data.

The overall aim of this paper is to better understand inequality in contextual poverty throughout space at different scales and to compare different residential locations as parts of an integrated urban system, and not as isolated spatial units. We use a multiscale approach to understand spatial inequality in contextual poverty in the Netherlands, by answering the following questions: Firstly, how ‘big’ is spatial inequality in contextual poverty in the Netherlands and where does it come from – from which spatial scales in which levels of the urban system (within and between municipalities)? Secondly, how, and to what extent, do different municipalities contribute to national level inequality – and at which spatial scales? Finally, how can we compare different spatial contexts if we know that they consist not only of a single spatial unit but of a range of spatial scales?

Methodologically, spatial scale is one aspect of the modifiable areal unit problem (MAUP; Manley, 2014; Openshaw, 1984), which suggests that measuring areal characteristics is affected by the size and exact boundaries of the spatial units. The variability of scales is, in our view, not a problem, but a reflection of multiple sociospatial processes and contexts in which people live, because phenomena not only can be potentially measured, but also exist at multiple scales (Manley et al., 2006; Petrović et al., 2019). Therefore, we operationalised multiscale contextual poverty as distance profiles, which include a range of 101 bespoke areas (centred on individual locations), representing people’s spatial contexts starting from very small neighbourhoods up to the city or regional level (see Petrović et al., 2018). These distance profiles then show for each location how potential exposure to poverty changes across spatial scale. For this, we used register data for the full population of the Netherlands, geocoded to 100 m by 100 m grid cells. To capture the complexity of spatial inequality, we used the Theil multilevel index of inequality (hierarchical entropy). Although entropy can be used to measure spatial inequality between units at a certain spatial scale, this paper goes further. It applies entropy at *multiple* spatial scales and uses it to measure inequality *across* different spatial scales in a single location. Using data for the whole of the Netherlands (100 m by 100 m grid cells), the study reveals what spatial scales of residential context are particularly relevant to better understand potential exposure to poverty in different municipalities in the Netherlands.

## Spatial Inequality in Contextual Poverty: The Issue of Spatial Scale

### The Measurement of Poverty

Although the literature generally distinguishes between absolute and relative poverty (George, 1980; Hagenaars, 2017), contextual poverty is relative in many ways: We need to define what poverty is within a certain frame of reference, and also to compare different areas. Poverty can be conceptualised and measured in many ways, but the most common concept is monetary poverty, whose indicator is an ‘at-risk-of-poverty rate’, i.e. the percentage of households or individuals with an equivalent net disposable income below a threshold (Goedemé & Rottiers, 2011). Indeed, relative income poverty measures should rather be regarded as indicators of poverty *risks* than of poverty per se (Bäckman, 2009). Since there is no universal measure of poverty, poverty can be considered an inherently relative, socially constructed concept (MacPherson & Silburn, 2002). One common way of measuring poverty is to set the threshold of the risk of poverty at a percentage of the national median income. Based on the work of ILO and OECD, this threshold is often two thirds of the median income (Goedemé & Rottiers, 2011; ILO, 2013; vom Berge et al., 2014), but lower cut-off points, such as those of 50% or 40% of the median, are also used (see Dixon & Macarov, 2002; Marlier, 2007; Bäckman, 2009). In global studies, the same absolute threshold can be used to compare countries (see Jen et al., 2009). Moreover, low-income can be defined as the lowest quartile, quintile, or other percentile of the income distribution (see, e.g., Hedman et al., 2015). In the variety of poverty definitions and low-income thresholds, the most important is to use an appropriate concept in a given geographic setting.

Besides the threshold of poverty risk, a fundamental issue is choosing the unit over which poverty is measured. Most empirical studies use the household as the smallest unit at which data are disaggregated (MacPherson & Silburn, 2002). Alternatively, individuals can be the unit of analysis, which may give different results, depending on household compositions. Due to the difference in size between richer and poorer households, the numbers of households and individuals below the poverty threshold may give different evidence of poverty incidence (Anand, 1983). Clearly, the definition of poverty will depend on the threshold of low income as well as on the basic unit of measure (individual or household) we used.

After we have selected a definition, spatial measures of poverty depend on the spatial structure we adopt. In the Netherlands, the major local administrative unit is municipality. In 2013, there were 408 municipalities, with an average population of 41,000 people and an average area of 100 km^2^ (Keuning, 2013). Municipalities can be urban, suburban, or rural, and they vary in area and populations, with almost half having between 20,000 and 50,000 inhabitants in 2013 (Keuning, 2013). However, the four most populated municipalities (Amsterdam, Rotterdam, The Hague, and Utrecht) had more than 250,00 inhabitants. To provide useful results for policy makers, it is important to consider the administrative division of space, in our case municipalities. Nevertheless, space is continuous and exposure to poverty occurs simultaneously at multiple spatial scales. Our study therefore does not ignore the administrative spatial structure, but it also does not impose administrative boundaries on exposure to spatial context.

### Exposure to Poverty from Macro to Micro Scale

Multiple spatial scales are important for understanding contextual poverty. Following Manley et al. (2015), we generalise them as macro, meso and micro scales, for a better understanding of the spatial context. There are, however, no universal definitions of any of these scales and we need to put them in a specific setting: in our case studying contextual poverty in the Netherlands. We can identify regional and city scales as relevant spatial contexts in which smaller neighbourhoods are embedded. These smaller neighbourhoods extend within a few hundred metres, while an area with a radius of few kilometres would already represent a considerable part of any middle-sized or larger Dutch city. While different spatial process are more specific to smaller or larger scales, all these scales are related.

Large-scale concentrations of poverty reflect regional economic structures and labour market conditions. Income inequality at very large scales, between countries and regions, has received a lot of attention in the economic and geographic literatures (Wilkinson & Pickett, 2006), because they help to understand economic performance, the cost of labour and housing, but also internal and even international migration. Therefore, most of the data are aggregated to large spatial units and many institutions which deal with causes or consequences of poverty work at the national or regional level. Although large-scale inequalities are in themselves important, what is missing is that they are rarely considered in relation to smaller spatial scales. Understanding metropolitan inequalities is necessary to fully understand neighbourhood-level mechanisms, as they represent an extralocal context of neighbourhood-level processes (Sampson, 2001). Large spatial scales, therefore, represent the ‘context of context’ for the small-scale neighbourhoods (Petrović et al., 2018). External contextual mechanisms result from a neighbourhood’s location relative to economic and political structures, for instance in terms of accessibility to jobs or public services (Horner, 2004; van Ham et al., 2001).

We relate concentrations of poverty within cities at the meso-scales to city-specific social, economic and housing characteristics. For example, many Western cities contain large urban districts composed predominantly of social housing (Bolt et al., 2010). These urban districts attract more low-income residents than areas with other types of housing, while the better-off residents often leave them (Bolt et al., 2009). Part of a city may develop a reputation based on its demographics or housing types, resulting in stigma by people from outside the neighbourhood, including potential employers (Taylor, 1998; White, 1998). Both the ‘objective’ quality of specific residential areas and their perceived reputation may affect decisions to move in to or out of the neighbourhood (Permentier et al., 2009; Sampson, 2012). These are all examples of processes operating at various meso-scales.

Exposure to poverty at small spatial scales influences people through social-interactive mechanisms, such as role models or social networks (Galster, 2012; Sampson et al., 2002). These mechanisms can, for example, impact on an individual’s job search behaviour, which often motivates studies on the effects of neighbourhood poverty on individual socioeconomic status (see, for example, Van der Klaauw & Van Ours, 2003). Although these studies normally refer to social-interactive mechanisms, they often use spatial units that are too large to capture these mechanisms. Furthermore, poverty in the micro spatial context is important at any level of social organisation of the population within the local community. Even in the absence of the local social organisation, neighbourhood as an immediate surrounding of home still remains an area of exposure (Sampson, 2001). Therefore, small spatial scales are necessary to operationalise proximity as a potential for exposure and contact in the residential context. At the same time, we should not forget that these micro spatial contexts are embedded within larger urban contexts.

Measuring spatial attributes at various scales from micro to macro generally gives different results, which is formulated as the modifiable areal unit problem (Openshaw & Taylor, 1979). However, altering spatial scale is more than a technical *problem* – it is a way to better understand the spatial context of people, from the immediate surrounding of their home up to a wider context of the city. This range of scales can be represented as a *distance profile*, which includes increasingly large areas around an individual’s residential location (Petrović et al., 2018). Therefore, distance profiles show how the residential context of an individual changes at different spatial scales. Simultaneously, depending on where they live (within a certain municipality, and in which municipality), different people have different spatial contexts at multiple scales.

### Inequality Within and Between Places

In all areas from very small neighbourhoods to urban regions, poverty can be better understood through the comparison with other areas: One neighbourhood is poorer than other neighbourhoods; during their life, people can move from a poorer to a richer part of the city; one region in the country is known for being better-off than other regions, e.g. for providing more opportunities for education and work. Poverty can, therefore, be analysed through the lens of spatial inequality. While a large body of literature studies inequality between countries (see, e.g. Bäckman, 2009), national policy makers are primarily concerned with inequalities between different places within a country.

Regional inequalities in the Netherlands exist between the largest cities and the rest of the country as well as between the core cities and suburbs. In the Netherlands cities are, on average, poorer than rural areas – in fact, the more inhabitants a municipality has, the larger is the proportion of low-income people, ranging from 7% in municipalities with less than 10,000 people, to 9% in municipalities with 50,000–100,000 people, up to 16% in municipalities with more than 250,000 people (four largest cities), in year 2012 (Vrooman et al., 2014). Almost a quarter of all low-income households lives in the four largest cities (Amsterdam, Rotterdam, The Hague, and Utrecht; see Vrooman et al., 2014). Focusing on specific urban regions, inequalities exist between core cities and their hinterlands. Van Kempen and Priemus (1999) warned that Dutch cities were moving towards a doughnut structure typical of American cities, where poverty concentrates in central cities, surrounded by relatively better-off suburbs. In the period 2004–2013, low-income households in the Amsterdam and Rotterdam urban regions increasingly moved towards the urban peripheries and surrounding regions, particularly to higher density satellite towns, directly or indirectly associated with the ongoing gentrification or the inner cities (Hochstenbach & Musterd, 2018). Despite this overarching trend of the suburbanisation of poverty, a growing number of working poor households remained highly urbanised, meaning that various processes are developing simultaneously.

In addition to higher proportions of low-income residents in big cities, these people are often spatially concentrated in specific neighbourhoods. Poor neighbourhoods are, therefore, found disproportionately in the larger urban areas, with almost 30% of the poor neighbourhoods, which are often targeted by urban policies, being located in the cities of Amsterdam, Rotterdam, The Hague, and Utrecht (Bolt & Van Kempen, 2003). Even within these neighbourhoods there are certainly further inequalities. However, many studies do not operationalise neighbourhoods at small spatial scales, largely due to the lack of data. Furthermore, notwithstanding the dominance of big cities, spatial inequalities remain important in smaller urban areas.

The above literature on the spatial patterns of poverty suggests that studies usually offer empirical evidence of spatial inequalities at singular scales, often because research focusses either on regional inequalities or inequalities between administratively defined neighbourhoods within specific cities. Combining consideration of both regional inequalities (between cities) and neighbourhood inequality (within cities), and even inequality within what is officially considered as ‘neighbourhood’, assists us understand which spatial scales matter more in specific cases. When large areas (e.g. two cities) are similar, micro locations stand out more. Likewise, when the differences between cities or regions increase, macro context more clearly determines the spatial footprint of inequality and consequently people’s individual life courses. This paper aims to understand contextual poverty through multiple spatial scales, including the full urban system, where small neighbourhoods are parts of larger urban areas. Inequity occurs between residential contexts of different people, living in different parts of their municipality, and different urban regions of the country. Crucially, the measurement starts from the premise that the spatial context of people changes as they move further and further from their home and this is how they experience inequality continuously in space.

## Data and Methods

To investigate different spatial scales of residential context, we created bespoke areas (centred around individual locations) at 101 scales. For this, we used individual level register data for the full population of the Netherlands, geocoded on 100 m × 100 m grid cells (Sociaal Statistisch Bestand – SSB, see Bakker, 2002; Houbiers, 2004), for the year 2013. Starting from each grid cell, bespoke areas spread in one hundred concentric circles, with radii ranging from 100 m up to 10 km with 100 m increments, to form a distance profile (for a more detailed description, see Petrović et al., 2018). The lowest scale – the 100 m × 100 m grid cell – represents an area of 0.01km^2^, while the largest ‘circle’ extends over 314km^2^. At all these spatial scales, we measured contextual poverty as the proportion of people with a low income (from work or social benefits) in 2013. To define low income, we used a relatively low threshold of below 40% of the national median income, given the high income level in the Netherlands compared to other countries. In doing so, we examine more extreme poverty in our geographical setting and focus on the within-country inequalities.

To compare poverty in different places and at different spatial scales, we used the Theil index (Theil, 1967). The Theil index is a hierarchical measure of entropy, enabling simultaneous comparison of areas at different levels of spatial organisation. The Theil index of *total* inequality, prior to its decomposition, measures how unequal the proportion of low-income people is for each bespoke area across the whole country. With 101 spatial scales, our approach resulted in 101 Theil indices:

$$\mathrm{T}={\sum }_{\mathrm{i}=1}^{\mathrm{n}}{\mathrm{s}}_{\mathrm{i}}\mathrm{log}({\mathrm{ns}}_{\mathrm{i}})$$

$${\mathrm{s}}_{\mathrm{i}}={\mathrm{y}}_{\mathrm{i}}/\sum_{\mathrm{i}=1}^{\mathrm{n}}{\mathrm{y}}_{\mathrm{i}}$$

- *n* = number of grid cells
- $y_i$ = proportion of low-income people for cell *i*, measured at specific scale

Since we are interested where this total inequality comes from and how different places contribute to national inequality, we compared the proportion of low-income people in the bespoke areas of the specific size *within* and *between* municipalities. We decomposed the Theil index of inequality at each spatial scale into its within and between components to see to which extent the inequality comes from differences between areas within the same municipality and to which extent the municipalities differ among themselves. The two inequality components are calculated as follows:

$$\begin{aligned}\mathrm{T}={\sum }_{\mathrm{g}=1}^{\upomega }{\mathrm{s}}_{\mathrm{g}}&\sum_{\mathrm{i}\in \mathrm{g}}{\mathrm{s}}_{\mathrm{i},\mathrm{g}}\mathrm{ln}({\mathrm{n}}_{\mathrm{g}}{\mathrm{s}}_{\mathrm{i},\mathrm{g}})+{\sum }_{\mathrm{g}=1}^{\upomega }{\mathrm{s}}_{\mathrm{g}}\mathrm{ln}(\mathrm{n}/{\mathrm{n}}_{\mathrm{g}}{\mathrm{s}}_{\mathrm{g}})\\ &{T_W} \qquad \qquad \qquad \qquad \qquad{T_B}\end{aligned}$$

$${\mathrm{s}}_{\mathrm{g}}=\sum_{\mathrm{i}\in \mathrm{g}}{\mathrm{y}}_{\mathrm{i},\mathrm{g}}/\sum_{\mathrm{i}}^{\mathrm{n}}{\mathrm{y}}_{\mathrm{i}}$$

$${\mathrm{s}}_{\mathrm{i},\mathrm{g}}={\mathrm{y}}_{\mathrm{i},\mathrm{g}}/\sum_{\mathrm{i}=1}^{{\mathrm{n}}_{\mathrm{g}}}{\mathrm{y}}_{\mathrm{i},\mathrm{g}}$$

- $n_g$ = number of grid cells in municipality *g*
- $y_i$ = proportion of low-income people for cell *i*, measured at specific scale
- $T_W$ = Within-municipality component of inequality
- $T_B$ = Between-municipality component of inequality

**Fig. 1:**
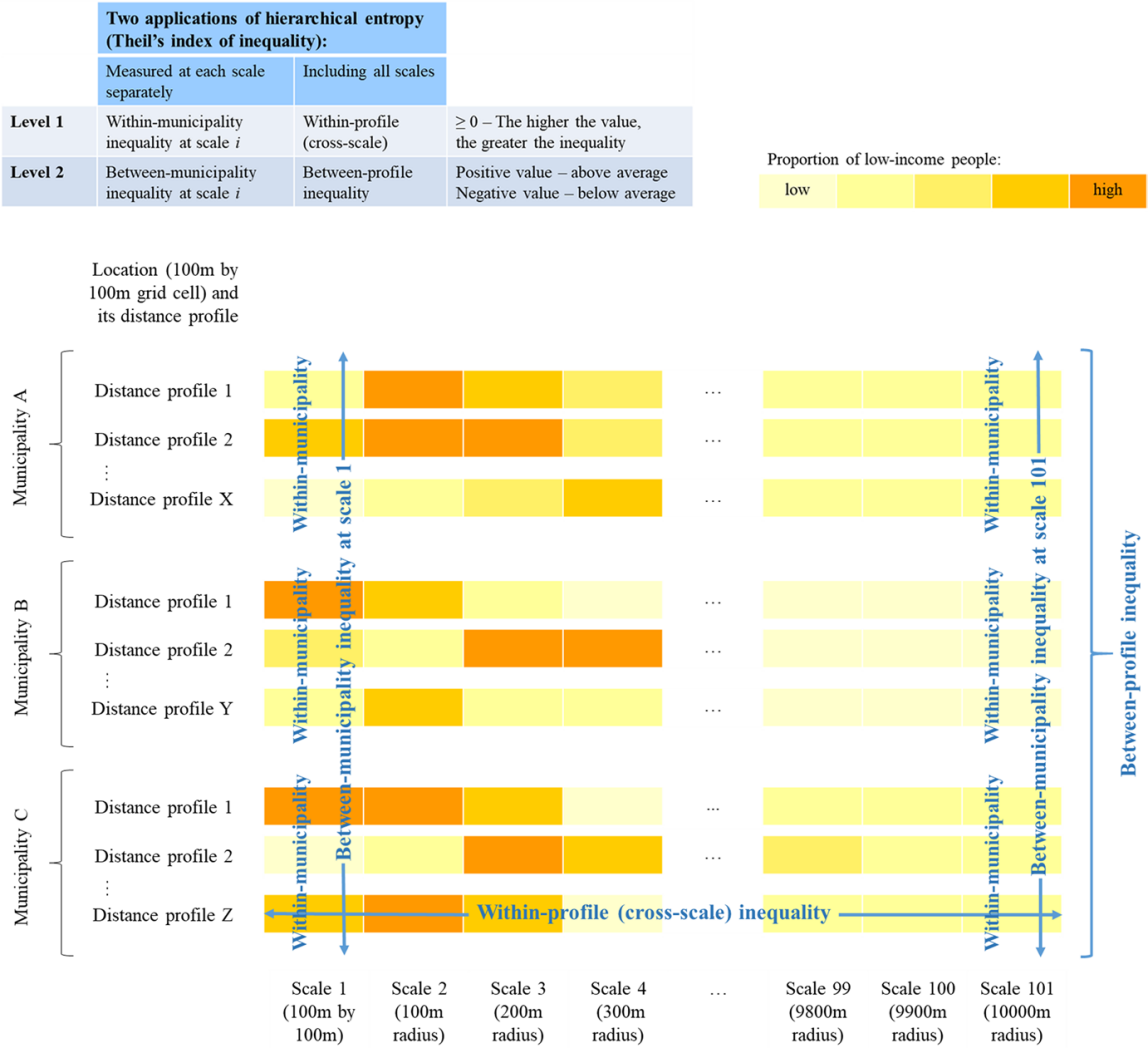
Two applications of the Theil index of inequality

Figure 1 illustrates this application of the Theil index using distance profiles in different municipalities. Municipalities A, B, and C represent a sample of three municipalities in the Netherlands, and the distance profiles (1 to X, 1 to Y, 1 to Z) have their starting points in a specific municipality, while the areas at larger spatial scales belonging to these profiles may expand outside the municipality border. At each scale, the first level of the hierarchical entropy measures inequality in the proportion of low-income people in different locations within municipalities (marked as ‘Within-municipality’ in Fig. 1). From this, we can observe how unequal the areas within municipalities are in terms of contextual poverty, where an index value of 0 means that they all have the same proportion of low-income people. From the second level of the index (that is, the between-municipality inequality), we can see if and by how much the areas within specific municipalities are above or below the national average in their potential exposure to contextual poverty. The between component of the index can, therefore, have both positive (indicating above national average) and negative values (below national average). These values provide a range of within- and between-municipality indices (one index for each scale), to demonstrate the effect of scale on measuring spatial inequality in contextual poverty.

However, entropy can give us more than this: it can also be used to measure scalar variability in the contextual poverty across spatial scales (Fig. 1). This is a less common use of entropy, which was demonstrated by Petrović et al. (2018), who used Shannon’s entropy to measure scalar variability in potential exposure to non-Western ethnic minorities. Here, we use Theil index to measure scalar variability in distance profiles encompassing the proportion of low-income people across 101 scales. This is the within-component of the index, measuring the inequality across scales within each distance profile, i.e. how the spatial context varies when we start from one location and include increasingly large areas (within-profile inequality). At the next level of the hierarchical entropy, we measure the inequality between the multiscale distance profiles, that is whether and to which extent they are above or below the national average in exposure to contextual poverty (between-profile inequality). In doing so, we compare locations not as single-scale units, but as multiscale spatial contexts. The elements in the Theil index equation are then the following:

- $n_g$ = 101 (number of scales in distance profile *g*)
- $y_i$ = proportion of low-income people at scale *i*
- $T_W$ = Within-profile (cross-scale) component of inequality
- $T_B$ = Between-profile component of inequality

## Results

To explore multiscale spatial contexts, we start from the smallest available scale and show the spatial distribution of low-income people in 100 m by 100 m grid cells. We then introduce other scales to get insight into how these various spatial contexts differ in terms of poverty levels, and to demonstrate the effect of spatial scale on measuring inequality. Finally, we encompass all the scales in one measure, showing the cross-scale patterns of the sociospatial inequality in the Netherlands.

### Spatial Distribution of Low-Income People at the Micro Scale

**Fig. 2:**
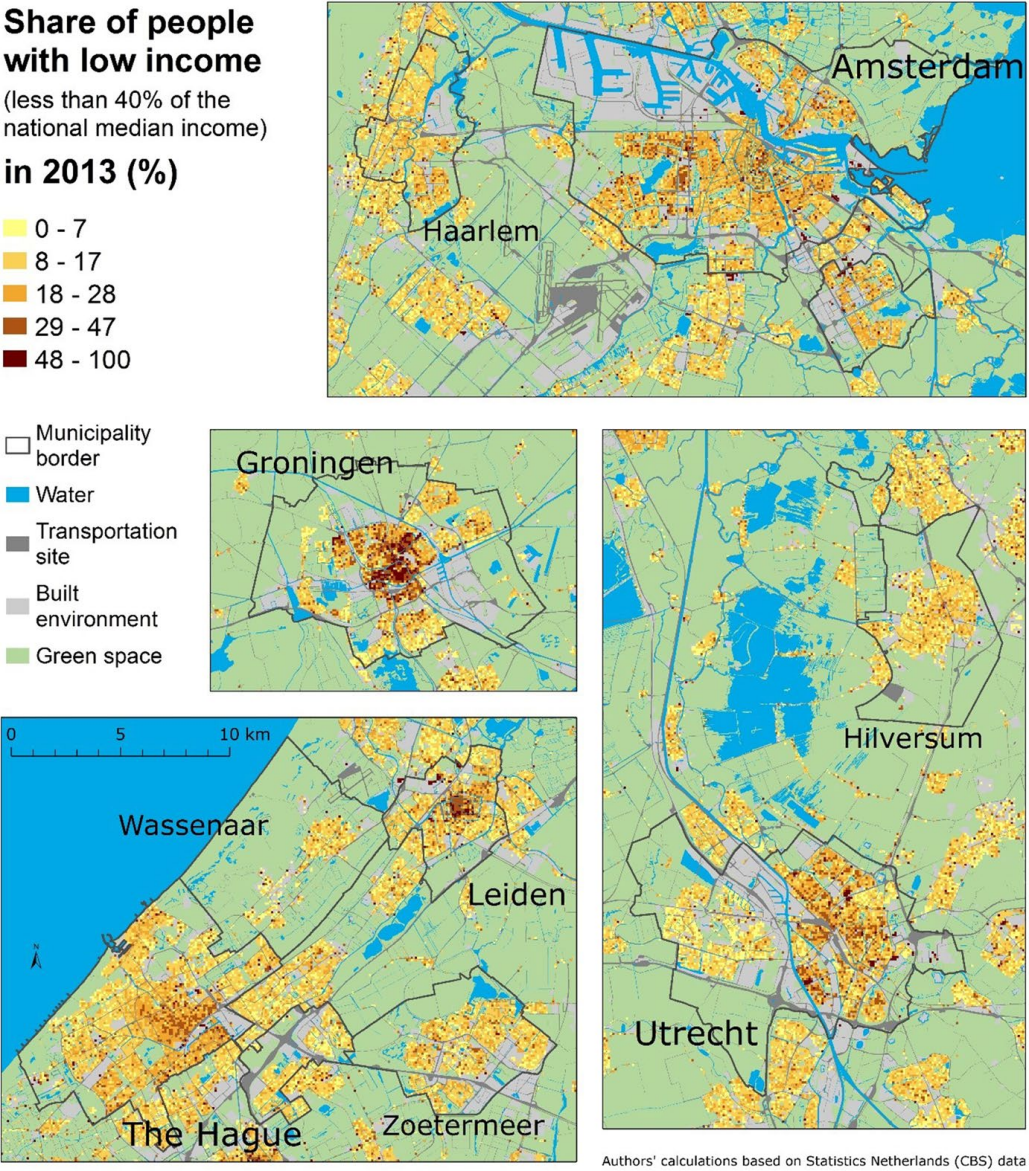
Proportion of low-income people in 100 m by 100 m grid cells in nine municipalities

Figure 2 shows the proportion of low-income people measured at the smallest available scale in nine sample municipalities, where low-income is below 40% of the national median. These municipalities present a mixture of places including some of the largest cities in the Netherlands (Amsterdam, The Hague, and Utrecht), as well as smaller nearby municipalities (Haarlem, Wassenaar, Zoetermeer, and Hilversum). However, it is notable that all of them are part of the Randstad, one of the largest conurbations in Europe. Additionally, we consider two middle-sized cities: Leiden (also part of Randstad) and Groningen, a relatively isolated city in the north of the Netherlands. The maps (Fig. 2) show that, in Amsterdam, low-income residents are scattered across the city. This may be related to the size and location of the social housing, of which there is a greater than average proportion in Amsterdam (third versus half respectively) and social housing is dispersed across the city (Musterd, 2014). In the other cities, particularly Groningen, low-income people are more concentrated in the city centre. Indeed, in Groningen a lot of small low-income neighbourhoods form the most striking concentration of contextual poverty among the selected municipalities. By contrast, smaller, peripheral municipalities of Haarlem, Wassenaar, Zoetermeer, and Hilversum have fewer low-income neighbourhoods, without obvious concentrations.

These maps give insight into the potential exposure to poverty in micro-neighbourhoods, a largely under-analysed scale of spatial context. However, for people it is also important to consider how their context changes throughout space. In many of these locations, the map would look differently if we delineated neighbourhoods at another spatial scale. Therefore, we need to complement the micro scale with the measures of contextual poverty at other scales in order to answer our first two questions: How big is the spatial inequality in contextual poverty in the Netherlands and where does it come from – from which spatial scales in which levels of urban system (within and between municipalities)? In which ways and to which extent different municipalities contribute to the national inequality – and at which spatial scales?

### Multiscale Spatial Inequality Within and Between Municipalities

**Fig. 3:**
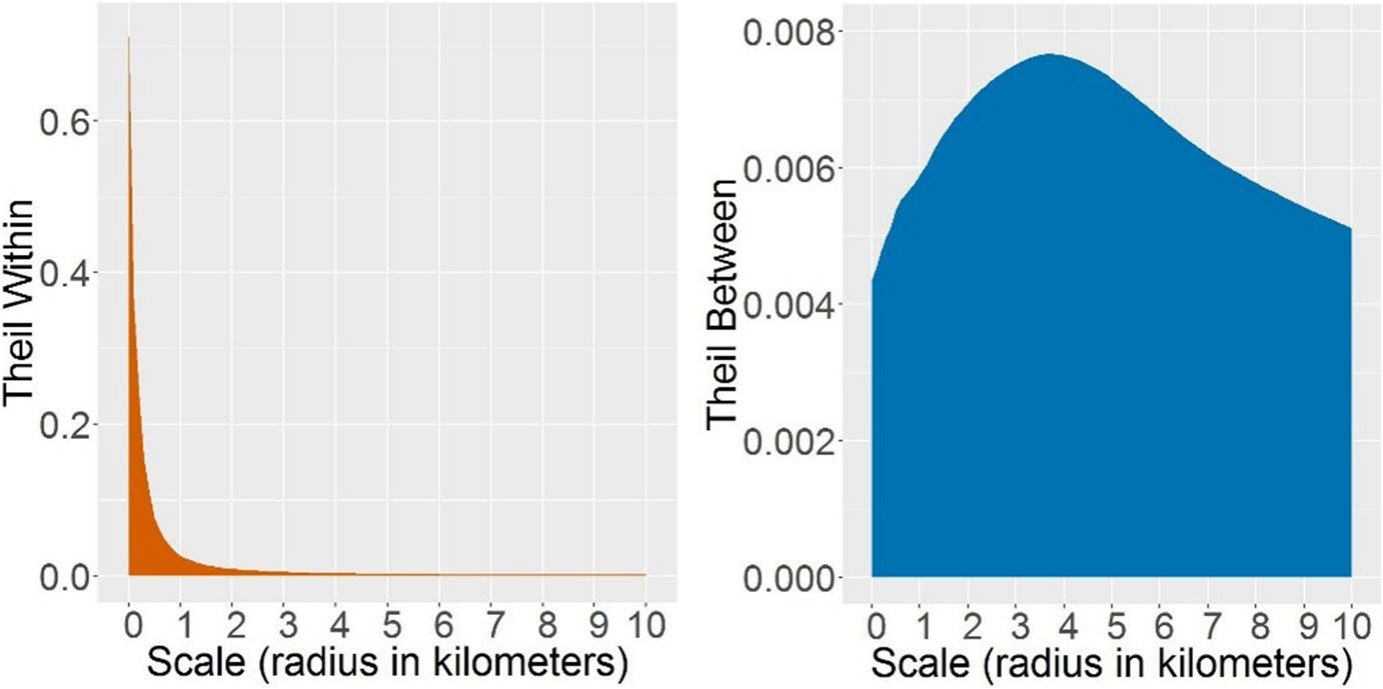
The Theil index of inequality in contextual poverty within and between municipalities at 101 spatial scales

To answer the first question regarding national inequality, Fig. 3 shows the Theil index of inequality in contextual poverty within and between all municipalities in the Netherlands, calculated separately for each of the 101 spatial scales. The left panel of the figure shows the inequity within municipalities, that is how different areas at the specific scale are in their poverty levels (spatial scale is shown on x-axis). The greater the value of the index, the greater is the within-municipality inequality. At the smallest scales (those under 1 km), there are big within-municipality differences. We would expect this to be the case because small neighbourhoods in a single municipality will be highly heterogeneous, ranging from those with a lot of poverty to the ones with very little or none. Moreover, at a fine spatial scale with relatively small populations, sharp differences can occur within a municipality. By contrast, very low inequality within municipalities occurs at the largest spatial scales, where all people in one municipality share similar contexts. The most critical aspect from the graph is that even for areas with a 2-3 km radius there are equal proportions of low-income people, which indicates that Dutch municipalities do not, in general, have large areas with distinct poverty levels.

Beyond this national pattern, there are, however, differences between municipalities – shown in the right panel of Fig. 3. Firstly, municipalities differ in potential exposure to poverty across all spatial scales, although at different extents. Secondly, the between-municipality index, which could be positive or negative, is constantly positive. This is because the national level of poverty is low, so that poverty levels cannot go much lower, but the municipalities with above-average levels of poverty stand out and push the index towards the higher positive values. Thirdly, there is a peak at the scale of 4 km. This means that, areas with a radius of 3-5 km appear to be the most appropriate for identifying concentrations of poverty in the Netherlands.

**Fig. 4:**
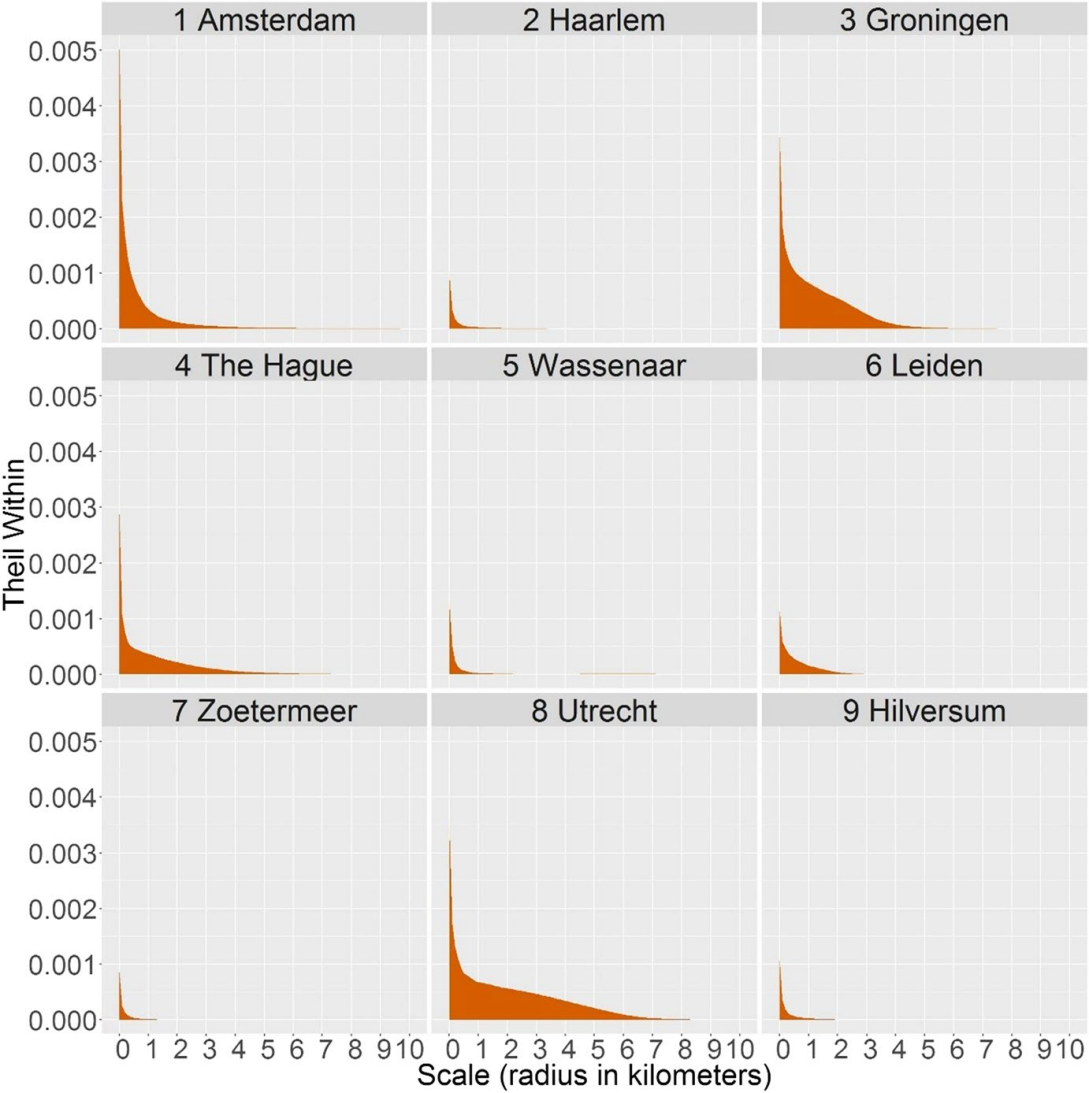
Contribution of nine municipalities to the Theil index of inequality in contextual poverty within municipalities at 101 spatial scales

**Fig. 5:**
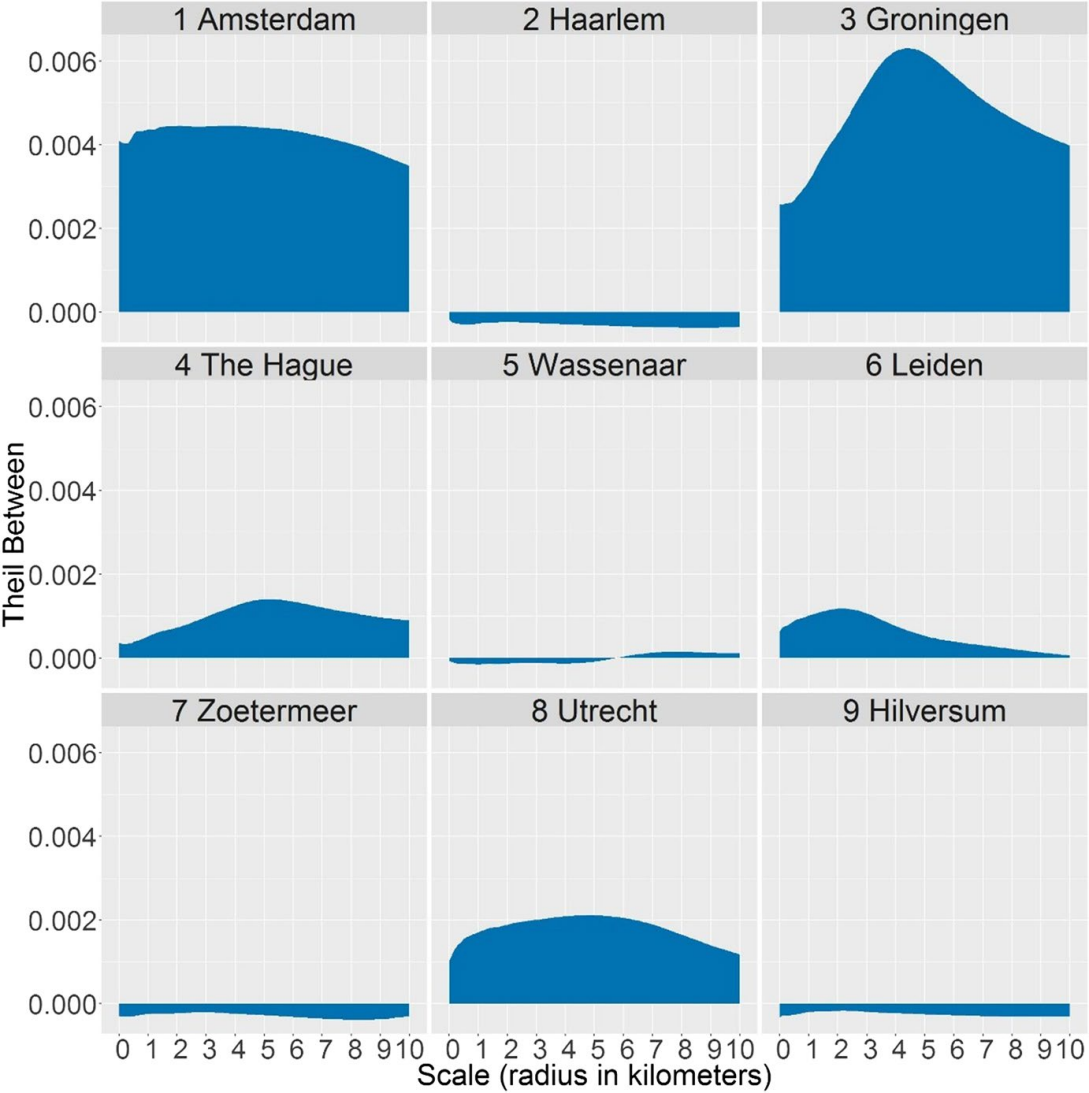
Contribution of nine municipalities to the Theil index of inequality in contextual poverty between municipalities at 101 spatial scales

The Theil indices (Fig. 3) include the data from all municipalities. However, each municipality contributes in a different way to the overall inequality, which leads us to our second question: How and to which extent different municipalities contribute to the national inequality – and at which spatial scales? Some municipalities have more diverse neighbourhoods than others (greater within-municipality inequality) and some have neighbourhoods with higher or lower poverty levels than neighbourhoods in other municipalities (greater – positive or negative – between-municipality inequality). The former is then measured by the within component of the Theil index; the latter – by the between component. Figures 4 and 5 show how the nine sample municipalities contribute to the overall inequality shown in Fig. 3.

Figure 4 shows the within-municipality component of the Theil index – that is, how unequal the areas are at various spatial scales within each of the nine municipalities. Increasing the scale at which we measure inequality helps us to identify how large areas with distinct proportions of low-income people are. For example, in Utrecht, Groningen, and The Hague, we can most clearly distinguish between parts of the city with unequal poverty levels, because the within-municipality persists up the scale of a few kilometres (in Utrecht even around 5 km). In Groningen, spatial inequality extends for areas up to around 3 km, but with a higher intensity than in other cities, which means that some areas (the city centre, see Fig. 2) have extremely high proportions of low-income people. On the contrary, Amsterdam has much less inequality at the meso scales, but has instead the greatest inequality of micro-neighbourhoods. Except Leiden, all larger and middle-sized cities have a great variety of neighbourhoods at the smallest spatial scales. The smaller municipalities of Haarlem, Wassenaar, Zoetermeer, and Hilversum have less inequality at all spatial scales, but their difference from the bigger cities is particularly visible at the smallest scales, where the small municipalities have much less diversity in neighbourhood poverty.

From Fig. 4, we saw that the municipalities differed in their internal inequality. The decomposition also allows to explicitly compare the municipalities and explore if their poverty levels at various spatial scales is above or below the national level. Figure 5, therefore, shows in which ways specific municipalities contribute to the national inequality between municipalities shown in the right panel of Fig. 3. The between component of the Theil index has a positive value if the municipality has more poverty than the national average, while a negative value occurs when it is lower. Firstly, larger cities generally have more poverty than smaller, more peripheral municipalities. Secondly, the figure shows at which spatial scales and to which extent poverty level in a specific municipality is different to the national average. Amsterdam, the largest city, has notably more poverty than the Netherlands on average, at almost all scales, while Groningen stands out with spatial concentrations of poverty within 4-5 km radii. The Hague has yet another pattern of scalar variability in contextual poverty: Small neighbourhoods fairly represent the national average in poverty levels, but the poorer ones tend to concentrate spatially, resulting in increasingly high index values.

Unlike bigger cities, the smaller, peripheral municipalities contribute to the national inequality mostly with negative values. This is particularly the case for Haarlem, Zoetermeer, and Hilversum, at the finest spatial scales, because the majority of small neighbourhoods have less poverty than the national average. It is also the case for the scales larger than 5 km, which often expand beyond the municipality border, and include parts of other municipalities. As such, Wassenaar is an interesting case of a small municipality with low overall poverty (hence negative values for the scales up to 6 km), but it is located between two cities with more poverty (The Hague and Leiden), and therefore has positive values at larger scales.

The case of Wassenaar demonstrates that neighbourhoods are not isolated spatial units; instead, they are parts of an integrated urban system. This also applies to the other municipalities: We can represent the increasingly large scales as a distance profile ranging from the small neighbourhood of 100 m by 100 m up to the area with a 10 km radius. Considering all the scales simultaneously, however, introduces additional complexity in comparing different residential locations. Our final question, therefore, is how we can compare different spatial contexts in terms of poverty, knowing that they do not consist only of a single spatial unit but of a range of spatial scales. So, the unit of analysis becomes the distance profile, consisting of 101 scales, and we use them for studying spatial patterns of poverty at multiple spatial scales simultaneously.

### Cross-Scale Patterns of Spatial Inequality

**Fig. 6:**
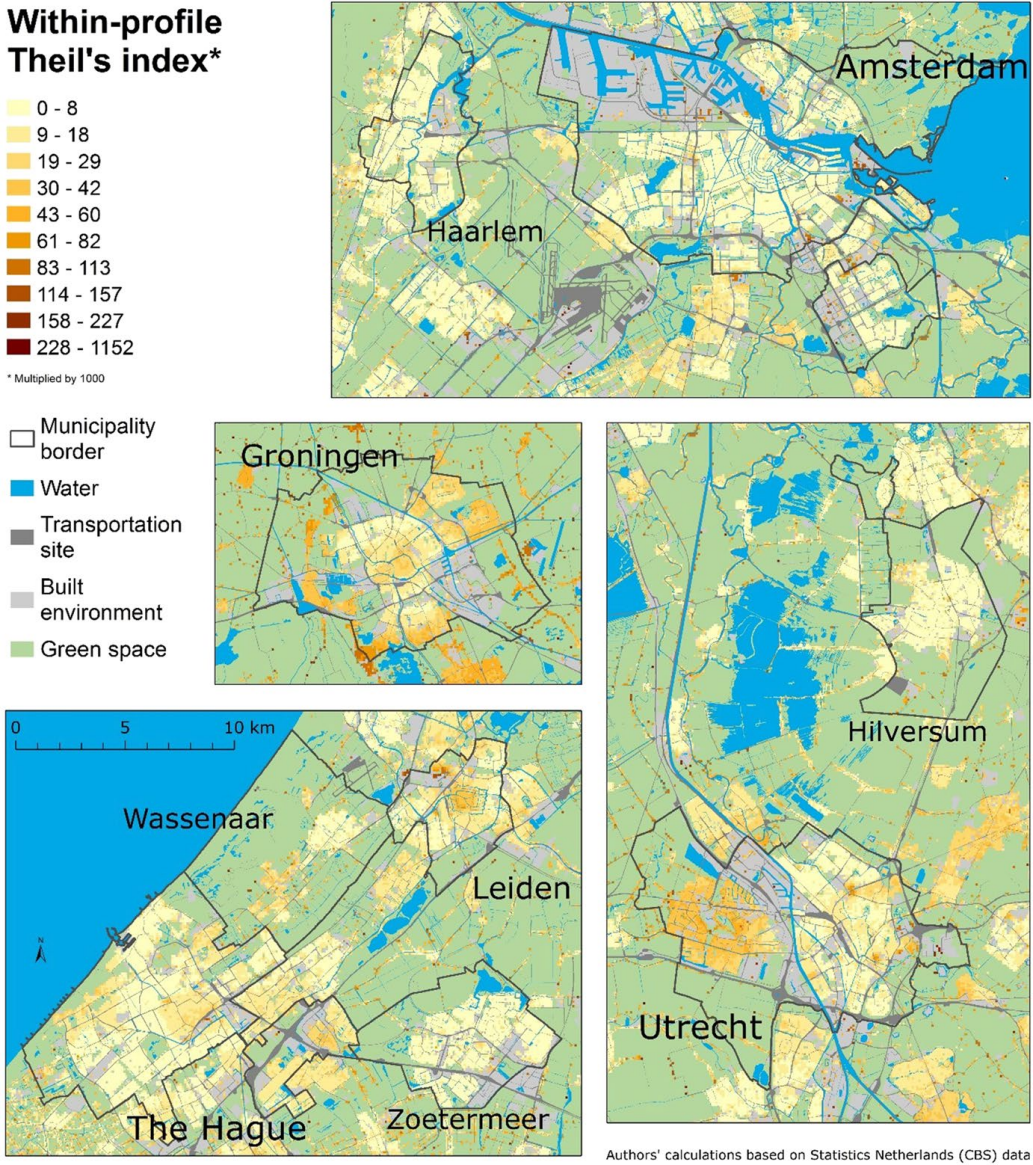
The Theil index of inequality across spatial scales within distance profiles in nine municipalities

**Fig. 7:**
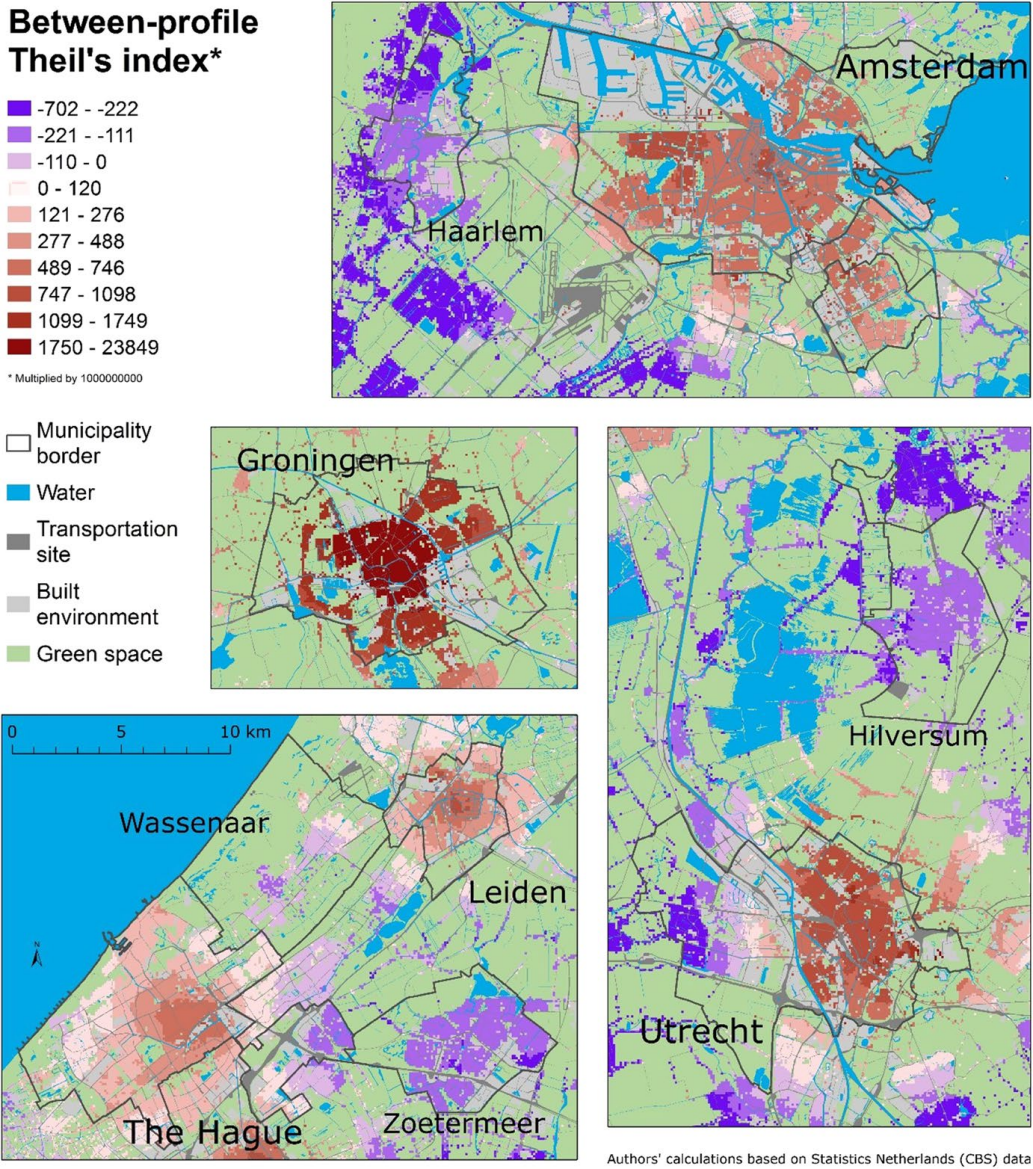
The Theil index of inequality between multiscale distance profiles in nine municipalities

To answer the final question, we decompose the Theil index – this time into the within-profile (cross-scale) inequality (Fig. 6), which is the scalar variability of distance profiles, and the between-profile inequality index (Fig. 7), which compares the distance profiles in terms of poverty levels at the range of scales. Figure 6 shows relatively low variability in most of the distance profiles. This is because low-income people are relatively scattered. The scalar variability of this socioeconomic group appears to be lower than for non-Western ethnic minorities, which have more clustered spatial patterns (see a comparable analysis by Petrović et al., 2018).

The exceptions of a greater cross-scale variability denote places with considerable spatial changes in contextual poverty. They are located, for instance, in the city centre and periphery of Groningen, because people are exposed to different spatial contexts as they move between these parts of the city. Another example is the western part of Utrecht, which is much more affluent than the larger, eastern part. These are both examples of greater variations at meso and macro scales, which result in greater cross-scale variability of the distance profiles than in other municipalities. In many residential locations in Leiden and The Hague, people are also potentially exposed to different levels of poverty as they move farther away from their home. In the rest of our study area – with the low within-profile variability – people’s spatial context does not vary considerably and persistently at a wider range of scales: it may vary at smaller scales and then stabilises. This is why the majority of residential locations with the very low within-profile index can be seen in Amsterdam – the city that we already identified as having the spatial inequality primarily at the lowest scales.

In addition to having different patterns of scalar variability, distance profiles vary in terms of their overall levels of poverty. Figure 7 shows how each distance profile, consisting of 101 scales, contributes to national inequality. Two colour ramps differentiate the direction as well as the intensity of the index. The shading, therefore, reflects the amount of poverty that people are potentially exposed by their place of residence, over all scales: Red (positive) areas score above the national poverty level, while purple (negative) areas score below. The difference between the positive and negative indices can be best explained using two extreme examples: If a poorer neighbourhood is surrounded by other poor neighbourhoods at a wider range of spatial scales, so that the contextual poverty is persistent across spatial scales, the distance profile will have a high, positive between-profile index (Fig. 7). Cells with such profiles are usually clustered in Groningen, since they share similar larger-scale surroundings, forming distinct pockets of multiscale contextual poverty. In contrast, if low poverty persists across a number of spatial scales, the profile has a low, negative index, and this means that people are exposed to little poverty in wider areas around their home. This can be seen in the smaller municipalities of Haarlem, Wassenaar, Zoetermeer, and Hilversum, with the exception of the northern neighbourhoods in Wassenaar, close to Leiden.

Figure 7 reports the spatial patterns at the regional level: smaller municipalities have less poverty than the nearby big cities, but more than their surrounding rural areas. We can see this from the direction and the intensity of the index across our entire study area: Haarlem, Hilversum, Zoetermeer, and a large part of Wassenaar score below the national poverty level (and also much lower than the big cities), but other, even smaller and more rural municipalities have even less poverty. Big cities are clearly characterised by multiscale contextual poverty. Among them there are, however, different spatial patterns: Contextual poverty in Amsterdam can be described as ‘multicentre’, although in the national comparison these centres are not so obvious. Utrecht is clearly divided into a poorer eastern part (larger part around the city centre) and the newer and more affluent western part of the city. Therefore, Utrecht is not uniformly more affluent than Amsterdam and Groningen, rather to one, spatially distinct, part of the city. The Hague, Leiden, and Groningen have another spatial pattern of poverty – the core-periphery distinction, where the city centre is poorer, persistently at multiple scales, than the more peripheral parts of the city.

## Discussion and Conclusions

In this study, we analysed spatial inequality in contextual poverty within and between places, focussing on a few big cities and smaller municipalities in the Netherlands. For each 100 m by 100 m grid cell, we measured the proportion of low-income people at the range of 101 spatial scales, and we used the Theil index as a hierarchical measure of entropy to measure inequality. The results showed that scale considerably influences the measurement of contextual poverty and consequently the comparisons of different places. Within- and between-municipality comparisons are crucial to understand contextual poverty, because we can only understand poverty in one area in reference to other areas within the same municipality and in other parts of the country. By considering the range of spatial scales within the framework of the administrative units (municipalities), the study connected the literature on the importance of exposure to poverty at multiple spatial scales on the one hand, and policy design that needs an administrative structure on the other hand.

Spatial scale is crucial to understand spatial inequality and this implies that policy measures should also be multiscale, and that different problems require different actions and interventions at different spatial scales. Given the great inequalities among very small neighbourhoods within municipalities, policies targeting contextual poverty should not simply rely on official neighbourhood definitions, overlooking the inner neighbourhood inequalities. National level polices should take into account that along with the within-municipality inequalities, there are considerable inequalities between municipalities: it might be the case that the scale of intervention that works in one city does not in another, even when they are both within the country and relatively closely located. Comparing all municipalities in the country, we identified the greatest poverty concentrations at the scales of 3-5 km. These are, therefore, the scales at which poverty studies and measures at the national level should seek to intervene.

The first application of the Theil index – measuring inequality within and between municipalities at multiple scales – revealed different spatial structures of neighbourhoods within the urban system. For example, while the micro neighbourhoods in The Hague did not considerably contribute to the national inequality (their poverty levels were about the national average), combined at meso scales they formed areas with above-average poverty levels in the national comparison. Changing inequality across spatial scale does not merely demonstrate the modifiable areal unit problem (MAUP); it adds to literature such as Manley et al. (2006) and Jones et al. (2018), highlighting the process nature of the MAUP – that different spatial scales capture different spatial processes. That these processes differ over space (as shown here) further highlights the need to identify the flexible geographies at multiple scales. We should also note that the processes vary over time as well. For example, smaller geographies reveal micro-concentrations of poverty, which have often been associated with social-interactive mechanisms of neighbourhood effects. Similarly, theory suggests that stigmatisation occurs at a larger spatial scale and our study has provided evidence on the spatial extent and location of areas which may be potentially stigmatised. At the largest scales, labour market factors, such as regional wage levels and migration of labour, become the most relevant. Here, our findings provide a basis for further investigations into spatial mismatch in labour markets.

Finally, various scales are parts of an integrated urban system. Therefore, the second application of the Theil index considered all scales of contexts in one location simultaneously. For an individual, inequality can be seen as a distance profile: some people potentially experience greater inequality because poverty levels change as they move further away from home. Furthermore, comparing different distance profiles showed that neighbourhoods differed not only in their own characteristics, but also that seemingly the same neighbourhoods may have different meso and macro contexts. Single spatial scale cannot give enough input for policy actions. Instead, various scales jointly define distinct areas of potential exposure to poverty and possible interventions.

The regional trend is that contextual poverty decreases as we go from big cities towards smaller municipalities, and further towards the surrounding rural areas. Although poverty is a prior concern of big cities, poverty in peripheral municipalities and middle-sizes cities in rural areas should not be lost out of sight. This study should generate more interest in the analysis of poverty in various urban (and rural) contexts. Using small increments in radius from the very micro to macro contexts allowed us to explore at a fine resolution the differences between locations, which revealed more detailed spatial patterns than when using fixed administrative boundaries. We particularly pointed out three different multiscale spatial patterns of contextual poverty – multicentre in Amsterdam, east–west in Utrecht, and core-periphery in The Hague, Leiden, and Groningen. This analysis expands our understanding on spatial patterns of poverty that were for a long time been characterised as poor inner cities and wealthier peripheries, and then as decentralisation and suburbanisation of poverty. Kavanagh et al. (2016) noted that the recent focus on the suburbanisation of poverty is debatable, because of the ambiguous definitions of suburban. We add to this that spatial patterns of poverty are more complex than usually presented, because the issue of spatial scale is often overlooked.

Measuring and understanding contextual poverty and its inequality over space largely depends on the spatial scale, because different spatial scales represent very different residential contexts. This is relevant for individuals who may be affected by the contextual poverty as well as for institutions at the local and national levels. Spatial scale may determine actions related to contextual poverty, because the same issue manifested at different spatial scale may require different solutions. Spatial scale also needs to be put in a certain context within the framework of spatial inequality, because differences occur both within and between places.
